# Coronary Microvascular Dysfunction in Patients with Congenital Heart Disease

**DOI:** 10.2174/1573403X19666230119112634

**Published:** 2023-07-05

**Authors:** Inne Vanreusel, Vincent F.M. Segers, Emeline Van Craenenbroeck, An Van Berendoncks

**Affiliations:** 1 Department of Cardiology, Antwerp University Hospital, Drie Eikenstraat 655, Edegem 2650, Belgium;; 2 Research Group Cardiovascular Diseases, GENCOR, University of Antwerp, Campus Drie Eiken, Universiteitsplein 1, Antwerp 2610, Belgium

**Keywords:** Microvascular (dys)function, coronary microvascular (dys)function, coronary flow reserve, microcirculation, heart defects, congenital heart disease

## Abstract

Congenital heart diseases represent a wide range of cardiac malformations. Medical and surgical advances have dramatically increased the survival of patients with congenital heart disease, leading to a continuously growing number of children, adolescents, and adults with congenital heart disease. Nevertheless, congenital heart disease patients have a worse prognosis compared to healthy individuals of similar age. There is substantial overlap in the pathophysiology of congenital heart disease and heart failure induced by other etiologies. Among the pathophysiological changes in heart failure, coronary microvascular dysfunction has recently emerged as a crucial modulator of disease initiation and progression. Similarly, coronary microvascular dysfunction could be important in the pathophysiology of congenital heart diseases as well. For this systematic review, studies on maximal vasodilatory capacity in the coronary microvascular bed in patients with congenital heart disease were searched using the PubMed database. To date, coronary microvascular dysfunction in congenital heart disease patients is incompletely understood because studies on this topic are rare and heterogeneous. The prevalence, extent, and pathophysiological relevance of coronary microvascular dysfunction in congenital heart diseases remain to be elucidated. Herein, we discuss what is currently known about coronary microvascular dysfunction in congenital heart disease and future directions.

## 1 INTRODUCTION

Congenital heart diseases (CHDs) represent a wide range of cardiac malformations [1]. Medical and surgical advances have dramatically increased the survival of patients with CHD, leading to a continuously growing number of children, adolescents, and adults with CHD [2, 3]. Nevertheless, CHD patients have lower physical fitness [3-5], reduced quality of life [6], and worse prognosis [7, 8] compared to healthy individuals of similar age. The development and progression of heart failure is the main cause of morbidity and mortality in this population [9, 10]. Patients with heart failure induced by CHD and patients with heart failure induced by other etiologies share many characteristics, including exercise intolerance [4, 11-13], (left and/or right) ventricular dysfunction [1, 14, 15], increased inflammatory cytokine levels [16], and neurohormonal activation [17].

Among the pathophysiological changes in heart failure, coronary microvascular dysfunction (CMD) has emerged as a key process [18]. For instance, in patients with heart failure, low-grade systemic inflammation triggers coronary endothelial inflammation and CMD [18], leading to structural and functional myocardial changes. The presence of CMD is associated with systemic endothelial dysfunction, (left and right) ventricular dysfunction [18], and reduced exercise capacity [19] and is a strong prognostic marker [20]. As CMD is an important driver of different types of heart failure, it is likely to be important in CHD as well.

**Fig. (1):**
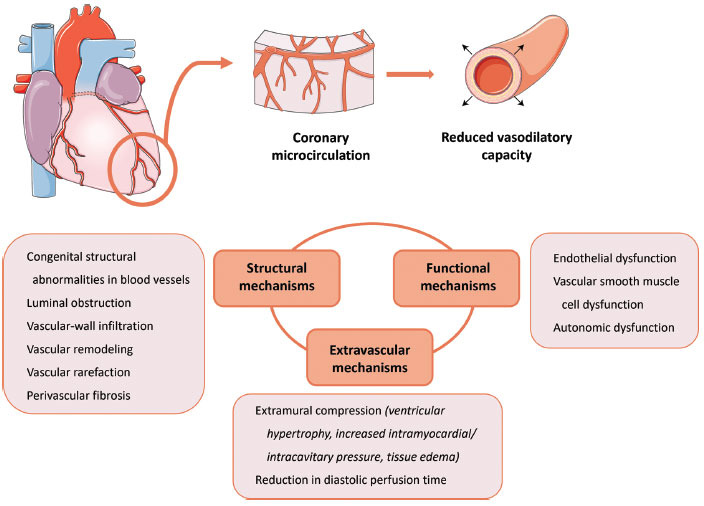
Different mechanisms of coronary microvascular dysfunction. The coronary microvasculature resides at the distal end of the coronary arterial system. CMD is a suboptimal coronary vasodilator response to exercise or pharmacological stress. Several pathophysiological mechanisms, including structural and functional alterations of the microvasculature as well as extravascular changes, account for CMD.

CMD can be defined as a suboptimal coronary vasodilator response to exercise or pharmacological stress [21]. CMD can be caused by several pathophysiological mechanisms, including structural and functional aberrations of the microvasculature as well as extravascular changes (Fig. **1**). CMD is not limited to dysfunctional endothelial cells but includes dysfunctional vascular smooth muscle cells, pericytes, and other cells in the microvessels. Different mechanisms may be important in different clinical scenarios, and more than one mechanism may be important in a particular scenario [22]. (For excellent reviews on this topic, kindly refer to [23-25]).

In clinical practice, the measurement of CMD relies on the principle of the coronary flow reserve (CFR), an integrated measure of coronary macro- and microvascular function [21, 26-28]. A CFR of less than 2.0 is considered abnormal [21, 29]. Measurement of coronary flow velocity and CFR by invasive coronary reactivity testing is widely regarded as the gold standard in the diagnosis of CMD [21, 30]. However, advances in cardiovascular imaging techniques have revolutionized the noninvasive detection of CMD.

The objective of this review is to gain more insight into coronary microvascular function in patients with CHD. Therefore, we review studies on the presence of CMD in both children and adults with CHD, as well as methods used to assess coronary microvascular function. Furthermore, we review factors associated with CMD and whether there are therapeutic options targeting CMD.

## 2 METHODS

Papers published in English were searched using PubMed. The search included the following keywords and respective MeSH terms: (((“Coronar*” OR “Coronary Vessels”[Mesh]) AND (“Microvascular” OR “Microcirculation”[Mesh] OR “Microvessels”[Mesh]) AND (“Function” OR “dysfunction”)) OR (“flow reserve”)) AND (“Congenital heart disease” OR “Heart Defects, Congenital”[Mesh]). This search identified 179 papers, which were screened based on title and abstract. To this end, the inclusion and exclusion criteria listed in Table **1** were used. Studies concerning coronary anomalies or myocardial bridging were excluded because macrovascular dysfunction could influence the interpretation of CMD. The referenced papers were also searched to identify additional studies. This review was finally based on 30 papers.

## 3 CMD IN CHD

### 3.1 Existing Literature on CMD in CHD

Studies on CMD in patients with CHD have been performed in broad patient populations consisting of multiple CHDs, as well as in narrow patient populations focused on one particular CHD. Most studies on CMD have been performed in patients with cyanotic heart disease, the majority in patients with transposition of the great arteries (TGA). Only four studies report results from patients with acyanotic heart disease. The study populations mostly consisted of children and young adults, although infants and adults were also examined in some studies. The main results of the literature search are summarized in Table **2**, which illustrates that, to date, most studies report a decreased CFR value in patients with CHD, indicating that CMD is common in CHD and important in its pathophysiology.

Three out of 30 papers concerned a review, of which two [30-34] concluded that coronary flow and CFR in children with CHD is a largely unexploited field and has vast potential for future research. The other papers consisted of 26 primary studies and one case report. A reduced CFR was reported in 20 studies [35-54]. In one paper studying children with TOF [55], reduced CFR values were found before surgery and in the early postoperative period, while CFR in the late postoperative period was significantly higher than in the earlier groups and did not differ from the CFR values in patients with Kawasaki disease [55]. CFR was not decreased in five studies, including a case report [56-60]. One paper did not compare CFR values with a control group but concluded that the site of coronary sinus drainage into the systemic atrium or the pulmonary venous atrium did not significantly affect CFR in patients long term after the Fontan operation.

Conflicting results exist within patient groups with a particular CHD; for example, one study [52] showed CMD after coarctation repair, whereas another study [56] did not.

It is important to mention that these studies used different measurement methods (Table **2**). Two studies validated coronary flow velocity (CFV) and coronary flow velocity reserve (CFVR) measurements by transthoracic echocardiography in pediatric patients with various heart diseases. The authors concluded that noninvasive measurement of CFV and CFVR in the proximal left anterior descending coronary artery (LAD) and the posterior descending artery (PDA) using transthoracic Doppler echocardiography accurately reflects the invasive measurement of CFV and CFVR by Doppler guidewire method in pediatric patients with various heart diseases [35, 36].

### 3.2 Methods to Assess CMD in Patients with CHD

Methods that have been performed to assess coronary microvascular function in patients with CHD are intracoronary Doppler guidewire, Doppler transthoracic echocardiography, myocardial contrast echocardiography (MCE), positron emission tomography (PET), and magnetic resonance imaging (MRI) (Table **2**). To induce maximal hyperemia, different vasodilators have been used. While it is well established that the normal adult heart can increase coronary flow by maximally 2.5 to 4 times the resting value [30, 60] and a CFR < 2.0 is often considered abnormal [21, 29], cutoff values of CFR values are dependent on, among other things, the method and vasodilator used [21, 30].

Adenosine was the most commonly used vasodilator in the studies reviewed here, but acetylcholine, papaverine, and dipyridamole were also mentioned. While these are all pharmacological triggers for the vasodilation in the coronary microcirculation [29], some are endothelium-dependent (acetylcholine), and others are endothelium-independent (papaverine, dipyridamole) vasodilatory substances [29]. Although adenosine is mostly classified as an endothelium-independent vasodilator [29], there is also literature stating that the vasodilator effect of adenosine is at least partially endothelium-dependent [61-63]. Many aspects are still not fully understood due to discrepancies [63]. However, the maximal blood flow response is both endothelium- and non-endothelium-dependent [29]. Moreover, evidence has been accumulating that not only endothelium-dependent vasodilation but also endothelium-independent vasodilation per se is impaired in individuals with cardiovascular risk factors and cardiovascular disease. Impaired endothelium-independent vasodilation is associated with structural vascular alterations and alterations in vascular smooth muscle cells [64]. Yates *et al.* [48] performed dipyridamole stress testing in children after Arterial Switch Operation (ASO) and suggested lower CFR in children compared to normal adult values, but the data in children must be interpreted with caution as it still has to be confirmed that dipyridamole is an effective pharmacological stress agent in children. One study [45] additionally evaluated coronary epicardial reactivity by measuring the coronary response to nitroglycerin, which potently dilate the larger coronary arteries but has only a minor effect on the smaller coronary arteries [64].

Some techniques (Doppler echocardiography and guidewire) use flow velocity values (cm/s), while others (PET, MRI, and MCE) measure absolute blood flow measurements (ml/min/g cardiac mass). For PC VEC MRI, the volumetric flow (ml/s) is calculated by the product of the ROI and its mean velocity for each stage [41, 56]. While the CFR values obtained by CFV are lower than those obtained by measuring increases in global myocardial perfusion [59], both are linearly correlated [37]. Although different terms can be used (respectively, coronary flow velocity reserve and myocardial (blood) flow reserve or myocardial perfusion reserve (index)), CFR is most commonly used.

Differences in the results of the publications could partly be due to their study of the different myocardial regions of interest. Doppler methods were used to examine either one or a combination of the coronary arteries or the great cardiac vein, which has smaller CFR values [53]. Measurements with PC VEC MRI are preferably performed in the coronary sinus rather than the coronary arteries [41]. With techniques assessing myocardial perfusion, it is possible to define different regions of interest. In their review of clinical applications of radionuclide imaging in CHD, Partington *et al*. [65] mentioned that in CHD, CFR is computed in the systemic ventricle [65]. While *in vivo* quantification of the MBF of the right ventricle is not reliable in healthy control subjects [50, 51], it should be feasible to compare myocardial flow parameters of pressure-loaded right ventricles and normal left ventricles [42, 49-51].

Some studies aimed to validate a specific method against the gold standard in patients with CHD. One study showed that noninvasive measurement of CFV and CFVR in the proximal LAD [36] and the PDA [35] using transthoracic Doppler echocardiography accurately reflects the invasive measurement of CFV and CFVR by Doppler guidewire method in children with various heart diseases. Another study [53] demonstrated that it was possible to measure the great cardiac vein (GCV) flow velocity and GCV flow velocity reserve in pediatric patients with transthoracic Doppler echocardiography in young children with left ventricular volume overload secondary to ventricular septal defect (VSD). There was a good correlation between the GCV and LAD flow velocity reserves (r = 0.73, *p* < 0.01).

### 3.3 Pathophysiology of CMD in CHD

To date, the exact pathophysiology underlying CMD in patients with CHD is unknown. CHDs cause multiple and distinct hemodynamic and functional changes that may directly or indirectly affect the coronary flow and myocardial perfusion, even despite anatomically normal coronary arteries [30, 34]. Both basal and maximal coronary flow can be affected and result in a reduced CFR [30, 34]. As CMD is a key underlying mechanism in different cardiovascular diseases, most notably heart failure, it is highly likely of importance in CHD as well.

As in other patient populations, several pathophysiological mechanisms, including structural and functional aberrations of the microvasculature as well as extravascular changes, disrupt the ability of the vessels to dilate and augment blood flow in response to increased tissue oxygen demand and thus account for CMD [21, 22] (Fig. **1**).

CMD can be caused by a number of structural mechanisms. These include inborn structural vascular abnormalities, vascular remodeling, capillary rarefaction, and perivascular fibrosis. Histology of extramural coronary arteries in cyanotic CHD showed loss of medial smooth muscle cells and increased medial collagen [39]. It is conceivable that these structural abnormalities, which have been identified in the media of the epicardial coronary arteries and great vessels [37], also exist in the smaller vessels in CHD patients. Brunken *et al*. [37] also demonstrated that remodeling of the coronary circulation compensates for the chronic rheologic changes, *e.g.,* increased viscosity, accompanying the systemic arterial hypoxemia observed in cyanotic CHD patients.

Depending on the nature of the congenital abnormality, the ventricles may be exposed to pressure overload [1], resulting in ventricular hypertrophy [1, 66]. In experimental models of pressure overload of either the left ventricle or the right ventricle, ventricular hypertrophy is associated with a decrease in myocardial capillary density [50], also known as capillary rarefaction. Indeed, the right ventricular myocardial microvascular density of the septal wall in TGA and tetralogy of Fallot (TOF) patients with right ventricular hypertrophy due to pressure overload is reduced. Moreover, the decreased capillary density appears to be related to a reduced myocardial perfusion reserve [43]. Hearts with hypoplastic left heart syndrome also show a reduction in the capillarization of both the right and left ventricles compared to age-matched controls [67]. Moreover, myocardial fibrosis, including perivascular fibrosis, is almost invariably present in all forms of heart failure and has also been observed in patients with CHD [14]. Fibrosis can also contribute to CMD in patients with CHD.

Besides structural mechanisms, extravascular mechanisms can affect myocardial perfusion as well, the most obvious being extravascular compression of arterioles and capillaries. Increased extravascular compressive forces, *e.g.,* caused by ventricular hypertrophy, can impair the ability of the coronary microcirculation to increase the blood flow in response to dilator stimuli [68].

A third major group of mechanisms underlying CMD is functional mechanisms. These include endothelial dysfunction, vascular smooth muscle cell dysfunction, and autonomic dysfunction. Polson *et al.* [69] presented data suggesting that infants with coarctation of the aorta already show signs of cardiovascular autonomic dysfunction. Moreover, the surgery itself can also alter the cardiac anatomy, hemodynamics, and autonomic function. For instance, extensive vascular surgery during the Norwood operation often leads to sympathetic denervation of the heart and potential inadequate cardiac reinnervation, similar to transplanted hearts [40]. Moreover, in patients with TGA after arterial switch operation (ASO), manipulation of the coronary arteries during surgery is thought to have some adverse effect on myocardial perfusion because tissue manipulation results in scar tissue and can also result in partial myocardial sympathetic denervation [44, 46]. Nevertheless, as the CFR was normal in patients after the Ross procedure, which also involves coronary reimplantation, the procedure of reimplantation alone could not explain the findings [46]. Moreover, CFR also seems to be impaired in unoperated patients with congenitally corrected transposition of the great arteries (ccTGA) and even to a similar extent than in patients after atrial switch operation (AtSO), again suggesting that the CMD cannot be explained only by the surgical insult [50].

**Fig. (2):**
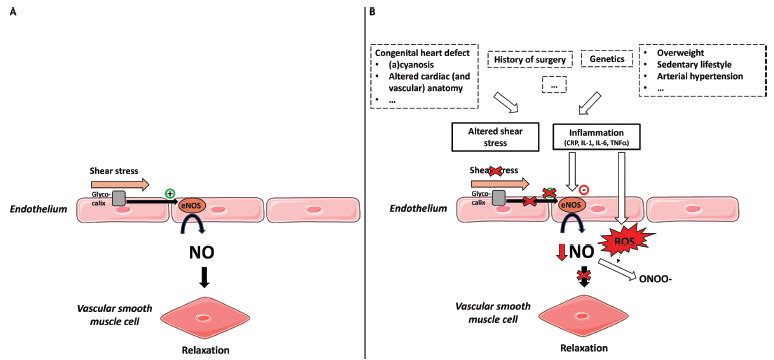
Hypothetical pathophysiology of coronary endothelial dysfunction in CHD. (**A**) Normal functioning of the coronary endothelial cell: shear stress affects multiple signaling pathways. Laminar shear stress, detected through deformation of the glycocalyx on the luminal side of the endothelial cells, results in activation of eNOS, resulting in nitric oxide production (98) and subsequently, relaxation of the vascular smooth muscle cells and vasodilation. (**B**) Hypothetical pathophysiology of coronary endothelial dysfunction caused by altered shear stress and inflammation: nonlaminar flow promotes changes to oxidative and inflammatory states of the artery wall (98). Proinflammatory cytokines may have a direct effect on endothelial cells, leading to depressed expression of eNOS, resulting in decreased NO synthesis (72). Moreover, in a pro-inflammatory state, endothelial cells reactively produce ROS. Production of ROS leads to the formation of ONOO-, also resulting in reduced NO bioavailability (99). **Abbreviations:** CRP: C-reactive protein, IL: interleukin, TNFα: tumor necrosis factor alfa, NO: nitric oxide, eNOS: endothelial NO synthase, ROS: reactive oxygen species, ONOO-: peroxynitrite.

CMD may also result from vascular smooth muscle cell dysfunction and/or endothelial cell dysfunction (Fig. **2**). Endothelial dysfunction has been studied intensively in heart failure, vascular disease, and CMD. Endothelial cells not only provide a barrier between the circulating blood and the underlying vascular smooth muscle cells and tissue cells, *e.g*., cardiomyocytes, but are also actively secreting numerous small molecules, peptides, and proteins [70]. Through the secretion of these paracrine factors, endothelial cells modulate the function of the underlying smooth muscle cells and cardiomyocytes.

Endothelial function can be affected by low-grade systemic inflammation. In analogy to the changes observed in heart failure [18], comorbidities might induce low-grade systemic inflammation, which triggers coronary endothelial inflammation and CMD. However, irrespective of comorbidities, there are several studies reporting elevated inflammatory markers in the blood of patients with a congenital heart defect, suggesting a low-grade proinflammatory state. For instance, a recent meta-analysis showed that serum levels of TNF-α in children with both cyanotic and acyanotic CHD were significantly higher than those in healthy controls [70, 71]. Moreover, in patients with successful coarctation repair, increased serum levels of proinflammatory cytokines and adhesion molecules (sICAM-1, sVCAM-1, E-selectin, IL-1b, IL-10, and sFas-ligand) were reported [72, 73]. Cardiac surgery also causes inflammation, as shown in a study on children with ventricular and atrioventricular septal defects; in these children, the concentration of CRP was elevated in the postoperative period after open heart surgery [34, 74].

The endothelium lining the cardiovascular system is highly sensitive to hemodynamic shear stresses present at the vessel luminal surface in the direction of blood flow. Normal laminar flow and shear stress can be altered by regions of flow disturbances near arterial branches, bifurcations, and curvatures but can also be altered by changes in local artery geometry during atherogenesis [75]. Therefore, one could expect that abnormalities in the cardiac and vascular anatomy in CHDs cause alterations in shear stress in the (coronary) arteries of these patients. In Fontan patients, for example, a common explanation for endothelial dysfunction is the lack of exposure of the pulmonary circulation to pulsatility and the associated decrease in the shear stress-mediated release of endothelium-derived nitric oxide [75-77], which substantiates this hypothesis. Moreover, secondary erythrocytosis, a physiological response to chronic hypoxemia in patients with cyanotic CHD, commonly results in increased whole-blood viscosity and shear stress, which may modify the balance between vasodilators and vasoconstrictors [38, 39, 78].

The increased blood viscosity is suggested as a trigger of compensatory vasodilation at rest in children with Fontan circulation [40] and as one of the causes of the observed higher basal flows in cyanotic CHD [37]. However, these increased blood flows at rest might also be influenced by other factors, including structural abnormalities of the vessels and increased myocardial oxygen demand caused by a chronically increased cardiac workload [37, 41] similar to acquired conditions that result in increased ventricular preload, ventricular mass, and volume overload [53]. For instance, patients with pre-Fontan pulmonary arterial banding, which causes myocardial hypertrophy, were found to have higher values for basal average peak velocities and significantly lower CFR values assessed by Doppler guidewire for both left and right coronary arteries than patients without banding [79]. Nevertheless, because an increased higher basal flow is not present in all patient groups, resting flow is probably not the only determinant of a reduced CFR [44]. The same applies to cyanosis because CMD is also observed in acyanotic heart defects.

Finally, genetic variants can also contribute to an increased susceptibility to CMD in CHD. For instance, a genetic variant of TNF-α (TNF-α rs1800629 A allele) was found to increase the susceptibility of CHD due to an increase in TNF-α expression [80]. Since TNF-α is an important inflammatory cytokine, this increase in TNF-α expression might also contribute to the development of endothelial dysfunction.

### 3.4 Clinical Relevance of CMD in CHD

While CMD is highly prevalent in association with adverse clinical outcomes in patients with various cardiovascular diseases [32], the significance and prognostic impact of a reduced CFR in CHD are currently unknown. In this section, we review factors associated with CMD in CHD. A summary of the published associations of different parameters with CMD testing in patients with CHD is presented in Table **3**. Here, we discuss in more detail ventricular function, exercise capacity, and peripheral endothelial function.

#### 3.4.1 Ventricular Function and Exercise Capacity

The positive associations between CFR/MBFR and ventricular function, as well as exercise capacity reported in patients with TOF [43] and ccTGA [49, 50], are of particular interest because these are two important outcome parameters. In contrast to these findings, however, neither blood flow at rest and during adenosine administration nor CFR were significantly correlated to gas exchange parameters from cardiopulmonary exercise testing, ejection fraction, and fractional shortening in TGA patients after ASO [46]. Also, in patients with successfully repaired coarctation of the aorta, CFR showed no significant correlation with left ventricular ejection fraction [56]. Furthermore, in patients after AtSO, the literature on associations with right ventricular function and exercise capacity is contradictory [43, 50]. The same applies to the relationship between ventricular function and CFR in patients with single ventricular morphology [42, 79].

**Fig. (3):**
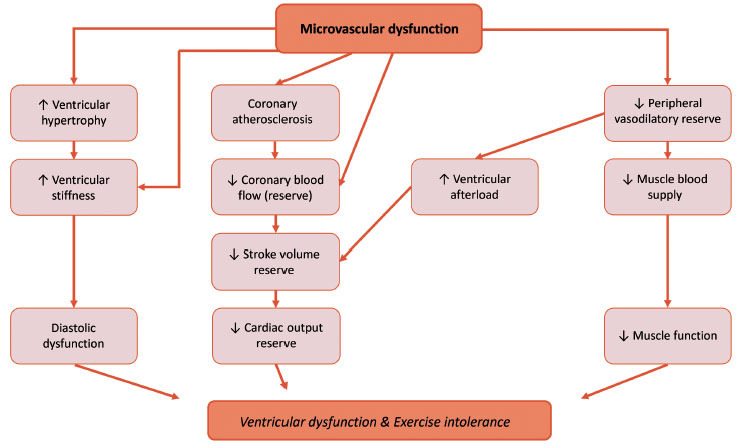
Hypothetical cardiac and noncardiac factors linking microvascular dysfunction to ventricular dysfunction and exercise intolerance in CHD. The “inflammatory microvascular dysfunction” hypothesis puts endothelial dysfunction at the root of ventricular hypertrophy and ventricular stiffness. Endothelial dysfunction is also a precursor of atherosclerosis. An important function of normal blood vessels is to vasodilate on exertion to meet the increased oxygen demands of skeletal muscles. This reactive vasodilation is regulated by shear stress on the endothelial cells. If one assumes that, as in HFpEF, CMD in CHD reflects a systemic disorder and consequently, peripheral microvascular dysfunction is present in addition to coronary artery disease, this vasodilation during exercise is disturbed, resulting in reduced muscle blood supply during exercise.

The relationship between CMD and ventricular dysfunction, as well as exercise intolerance in CHD, is most likely bidirectional. Analogous to CMD in patients with heart failure [81], CMD in patients with CHD might contribute to ventricular dysfunction and exercise intolerance through modulation of cardiac function and noncardiac disturbances (Fig. **3**). Due to the overlap in myocardial remodeling seen with other forms of heart failure, it can be hypothesized that endothelial dysfunction is a key mechanistic driver of ventricular dysfunction in CHD.

The relationship between ventricular dysfunction and exercise intolerance is straightforward: The capacity for performing aerobic exercise depends on the ability of the heart to increase cardiac output [5]. In patients with systemic right ventricle [82] and patients with Fontan circulation [83], ventricular function correlates with maximal exercise capacity. Correspondingly, the positive impact of exercise training on both left and right ventricular and systolic and diastolic function has been established [84, 85]. Nevertheless, to the best of our knowledge, such a beneficial effect of exercise training on ventricular function in CHD has not yet been investigated.

As mentioned earlier, depending on the nature of the congenital abnormality, the ventricles may be exposed to pressure overload, resulting in ventricular hypertrophy and subsequently reduced microvascular density and extramural compression. One could also expect that abnormalities in the cardiac and vascular anatomy and function in CHDs cause alterations in shear stress in the (coronary) arteries of these patients. Consequently, it seems logical that ventricular dysfunction may also contribute to CMD in CHD.

It is known that physical inactivity increases oxidative stress, endothelial dysfunction, and atherosclerosis [86]. Moreover, in other cardiovascular disorders, exercise is known to reduce inflammation and improve endothelial function [81, 85, 87, 88]. To date, the effect of exercise training on CMD in CHD has not been studied.

#### 3.4.2 Peripheral Endothelial Function

In Fig. (**3**), the assumption is made that, as in specific forms of heart failure, CMD in CHD reflects a systemic disorder. Two studies [41, 56] performed on CHD patients also examined, besides CMD, peripheral endothelial function by flow-mediated dilation (FMD), and in both patient groups (patients who have undergone TOF repair [41] and a selected group of coarctation-corrected patients [56]), FMD was normal. There was also no statistically significant correlation between FMD and CFR (Table **3**). Nevertheless, FMD is a method for assessment of the conduit arteries instead of resistance vessels. At present, no link has yet been established between coronary and peripheral endothelial function at a microvascular level in CHD patients [18].

## 4 FUTURE PERSPECTIVES AND LIMITATIONS

CMD has been shown to be crucial in the pathophysiology of different forms of heart failure. Our current knowledge of CMD in CHD patients, however, is more limited. The prevalence, extent, and pathophysiological contribution of CMD in CHD remains to be elucidated. As CHD shares many pathophysiological mechanisms with other forms of heart failure, it can be expected that CMD is important in CHD and could represent a target for therapeutic interventions to increase both the quality of life and survival of patients with CHD.

This review summarizes the available literature on coronary microvascular (dys)function in CHD. Performing a meta-analysis was not possible due to heterogeneity between studies. First, the studies focused on different CHDs. Second, even within a single CHD, comparison between studies was often difficult because of differences in patient characteristics (age group, surgical history, medication intake, and presence of comorbidities, among others). Third, the studies used various techniques with different vasodilators to assess microvascular function. Consequently, the values obtained often cannot be compared directly. Fourth, some studies included a control group, while others compared the results of CHD with existing literature. Due to ethical restraints, the control group did not always consist of healthy volunteers, and reference values from completely healthy controls were not always available [89].

Direct assessment of microvascular flow and morphology of the coronary microcirculation is complex due to the small size of the coronary microcirculation and the limited resolution of available imaging techniques. Therefore, assessment is usually based on evaluating its physiological and functional properties [21, 22]. The use of CFR as an indicator of coronary microvascular function is contingent upon the absence of epicardial coronary artery disease [22]. Therefore, without first excluding epicardial coronary disease or abnormality, CFR cannot be used to measure only the coronary microvascular function. Some studies reported on epicardial coronary artery assessment, *e.g*., the presence or absence of coronary artery anomaly, atherosclerosis, or stenosis and whether or not perfusion defects are present, which was only the case in a small minority. In the other studies, however, nothing is specified about the epicardial coronaries. On the other hand, most studies were conducted on children or young adults, a population with a low prevalence of atherosclerosis.

In general, the existing literature on CMD in CHD is inconclusive for several reasons. First, the numbers of studies conducted on the same type of heart defect are limited. Second, the small sample size of these studies is small, which is common in studies involving disorders with a low prevalence [9], making it hard to draw solid conclusions. Third, the results are often conflicting. Fourth, if coronary microvascular function appeared to be abnormal, the underlying mechanisms were often unknown. Nevertheless, associations were explored in some studies, for example, with parameters of ventricular function and exercise capacity.

Several pathophysiological mechanisms, including structural and functional aberrations of the microvasculature as well as extravascular changes, disrupt the ability of the vessels to vasodilate and augment blood flow in response to increased tissue oxygen demand and may therefore account for CMD [21, 22]. Different mechanisms of CMD could be involved in different CHDs. Moreover, since CFR is a ratio, it is not only affected by conditions influencing maximal blood flow but also dependent on the flow velocities at the basal state, making it even more difficult to compare results. As in patients with heart failure, CMD may be important in the pathophysiology of CHD and could influence cardiac remodeling, disease progression, and prognosis.

To date, no link has yet been established between coronary and peripheral endothelial function at a microvascular level in CHD patients, as is the case for other forms of heart failure [18]. Moreover, no studies have been published on treatment options targeting CMD in CHD. Furthermore, while CMD is highly prevalent in association with adverse clinical outcomes in patients with various cardiovascular diseases [32], the significance and long-term prognostic impact of a reduced CFR in CHD are currently unknown [90-99].

## CONCLUSION

To date, coronary microvascular dysfunction in congenital heart disease patients is incompletely understood because studies on this topic are rare and heterogeneous. The prevalence, extent, and pathophysiological relevance of CMD in CHD remain to be elucidated.

## Figures and Tables

**Table 1:** Inclusion and exclusion criteria for papers in this review.

**Category**	**Inclusion Criteria**	**Exclusion Criteria**
*Patient population*	Children and adults with CHD	Presence of macrovascular coronary artery disease Coronary artery anomaly or myocardial bridging Research performed on animals
*Assessment * *method*	Assessment of the maximal vasodilatory capacity in the coronary microvascular bed: coronary vasodilator response to exercise or pharmacological stress	Assessment of exclusively macrovascular vasodilatory function *e.g.,* fractional flow reserve Only baseline measurements or response to vasoconstrictors. Coronary diameter and baseline flow are examples of baseline measurements
*Article*	Articles in English	No full-text article is available Letters to the editor

**Table 2:** Summary of studies on coronary microvascular function testing in patients with CHD.

**Pathology**	**Number of ** **Articles**	**Age Group**	**Surgery**	**Controls**	**Measurement Method**	**Conclusion (↔ Controls)**	**Reported CFR Value in CHD**	**References**
**Children with CHD (Cyanotic and Acyanotic)**								
**CHD**	2	Children	NS	Kawasaki	Doppler TTE & Doppler guidewire (adenosine)	*Doppler TTE validated* CFVR reduced	*Calculation self-performed (only possible for LAD)*: 1.67 ± 0.10 (TTE) 1.72 ± 0 0.21 (guidewire)	[35, 36]
**Cyanotic CHD**								
**CCHD**	3	Adults	NS	Healthy	PET (dipyridamole)	MPR/CFR reduced	2.31 ± 0.92 in LV 2.08 ± 0.84 in septum 2.17 ± 0.77 in RV	[37-39]
**Single ventricle**	4	Infants	Yes (Norwood palliation for HLHS)	Infants after surgical repair of structural CHD	PET (adenosine)	CFR comparable	1.60 ± 0.28 in RV (only flow to the systemic ventricle was measured)	[57]
		Children	Yes (in Fontan circulation)	Healthy	CMR (adenosine)	MBFR was lower in HLHS, but once rest, MBF was normalized* by RPP: comparable	2.59 ± 0.78 *1.63 ± 0.53 (in the systemic RV)	[40]
		Children - young adults	Yes (Fontan-like operation)	Healthy	PET (adenosine)	CFR reduced	2.50 ± 0.88 (global MBF, with ROI in the systemic ventricular free wall)	[42]
			Yes (Fontan operation)	CS draining into pulmonary ↔ systemic venous compartment	Doppler guidewire (papaverine)	Site of coronary drainage did not significantly affect CFR	*Calculation self-performed:* RCA: 3.8 ± 1.04 (systemic 4.1 ± 0.8, pulmonary 3.4 ± 1.2) LCA: 3.9 ± 1.7 (systemic 4.2 ± 1.6, pulmonary 3.6 ± 1.7)	[79]
**TOF**	3	Children	Pre- and post-operatively	Kawasaki	Doppler guidewire (adenosine)	CFR lower pre- and early post-operatively; CFR normal late postoperatively	Pre-op: LAD 1.88 ± 0.57 RCA 1.85 ± 0.58 Early: LAD 1.60 ± 0.41 RCA 1.72 ± 0.48 Late: LAD 2.82 ± 0.83 RCA 2.95 ± 0.58	[55]
		Young adults	Yes	Healthy	PC VEC MRI (adenosine)	CFR reduced	1.19 ± 0.34 in CS	[41]
		Adults	Yes	Healthy	MCE (adenosine)	MBFR reduced	3.37 ± 1.57 in septal region (3.37 ± 2.04 in RV-free wall - not measured in controls)	[43]
**TGA**	11 + 1 case report†	Children – young adults	Yes (ASO)	Healthy	PET (adenosine)	CFR reduced	Global MBFR: 3.0 ± 0.6 in simple/early TGA, 2.9 ± 0.6 in complex/late TGA	[44]
			-	Literature: normal adults [90]	PET (dipyridamole)	-	1.19 ± 0.14 (mean regional MBFR)	[48]
				Healthy & Ross	PET (adenosine)		2.54 ± 0.61 (global MBFR)	[46]
				Healthy	Doppler guidewire (adenosine, acetylcholine)		Adenosine: 2.7 ± 1.5 Acetylcholine: 2.3 ± 0.9	[45]
				Literature: post-ASO patient with stenosis [91], Kawasaki [92, 93]	Doppler TTE (adenosine)		1.91 ± 0.51	[47]
				Literature: healthy children and adults [94-96]	Doppler guidewire (adenosine)	CFR comparable	3.7 (range 3.0-4.8) in LAD 3.4 (range 2.9-4.8) in RCA	[59]
				Literature: Oskarsson *et al.* [59]	Doppler guidewire (papaverine)		3.9 in LCA 3.4 in RCA	[58]†
				*Literature: near-normal children* [94]	PET (adenosine)	MFR comparable	3.51 (range 1.32-5.54) (global MBFR; also analyzed for each coronary territory)	[60]
			Yes (AtSO)	Healthy	PET (adenosine)	MFR reduced	2.93 ± 0.63 (ROI in the systemic ventricular free wall)	[51]
						CFR reduced	2.3 ± 0.6 (global MBFR of systemic RV)	[50]
					MCE (adenosine)	MBFR reduced	2.68 ± 1.13 in septal region (3.45 ± 1.35 in RV-free wall – not measured in controls)	[43]
			No (ccTGA)	Healthy	PET (adenosine)	CFR reduced	2.6 ± 0 .4 (global MBF of systemic RV)	[50]
							2.5 ± 0.28 in isolated ccTGA 2.6 ± 0.48 with associated anomalies (global MBF of systemic RV)	[49]
**Acyanotic CHD**								
**VSD**	1	Young children	NS	*Literature: normal children* [97]	Doppler TTE (adenosine)	CFVR reduced	1.58 ± 0.19 in GCV 1.77 ± 0.17 in LAD	[53]
**CoA**	2	Adolescents -young adults	Yes	Healthy	CMR (adenosine)	MPRI reduced	0.75 ± 0.22	[52]
					PC VEC MRI (adenosine)	CFR comparable	1.98 ± 0.38 in CS	[56]
**AS (+ BAV in 10/11)**	1	Infants/ children	No	Children with hemodynamically trivial CHD	CMR (adenosine)	Maximal MBF reduced, basal MBF comparable	MBFR values not calculated	[54]

**Abbreviations:** AS = aortic stenosis, ASO = arterial switch operation, AtSO = atrial switch operation, CF(V)R = coronary flow (velocity) reserve, (C)CHD = (cyanotic) congenital heart disease, CMD = coronary microvascular dysfunction, CMR = cardiac magnetic resonance, CoA = coarctation of the aorta, CS = coronary sinus, CTA = computed tomography angiography, GCV = great cardiac vein, HLHS = hypoplastic left heart syndrome, LAD = left anterior descending coronary artery, LCA = left coronary artery, LV = left ventricle/ventricular, MCE = myocardial contrast echocardiography, M(B)F(R) = myocardial (blood) flow (reserve), MPR(I) = myocardial perfusion reserve (index), NS = not specified, PC VEC MRI= Phase-contrast velocity encoding cine magnetic resonance imaging, PET = positron emission tomography, RCA = right coronary artery, RPP = rate-pressure product, RV = right ventricle/ventricular, (cc)TGA = (congenitally corrected) transposition of the great arteries, TOF = tetralogy of Fallot, TTE = transthoracic echocardiography, VSD = ventricular septal defect.

**Table 3:** Summary of the correlations/associations with coronary microvascular function testing in patients with congenital heart disease.

**Pathology**		**Outcome Measure**	**Correlations/associations**			**References**
			**Positive**	**Negative**	**Not Statistically Significant**	
**Single ** **ventricle**		CFR	Systolic ventricular function (cut-of: EF>0.50, EF<0.45)	Right (↔ left) ventricular morphology of the systemic ventricle	Site of CS drainage, age at operation and preoperative time interval of cyanosis, wall stress, cardiac index/end-diastolic/ pulmonary artery/systemic/pulmonary venous pressure	[42]
			Pulmonary arteriolar resistance (with RCA-CFR)	Pre-Fontan pulmonary banding*	Site of CS drainage, coronary sinus pressure, age at operation, time between Fontan operation and examination, pulmonary/systemic blood flow, systemic vascular resistance, mixed venous oxygen saturation, mean pulmonary arterial pressure, mean right/left atrial pressure, transpulmonary pressure gradient, cardiac index, EF, ventricular volume index, and coronary fistula	[79]
**TOF**		CFR	-	-	FMD	[41]
		MBFR	TAPSE, exercise capacity (% of predicted workload)	-	-	[43]
**TGA**	ASO	CFR	-	-	Simple TGA + early one-stage repair = more complex anomalies + later repair Aortic cross-clamp time, total bypass time at the surgery	[44]
			-	-	Aortic cross-clamp time, total bypass time at surgery, gas exchange parameters from CPET, EF, FS, levels of glycogen phosphorylase isoenzyme BB/creatinine kinase/troponin T	[46]
			-	-	Associated VSD, preoperative coronary anatomy, timing of the arterial switch operation, time since surgery	[47]
			-	Resting APV in RCA (with RCA-CFR)	-	[59]
		MFR	-	Anatomic LAD abnormalities†	Coronary artery z score [proximal coronary artery diameters]	[60]
-	AtSO	CFR	-	-	ACE inhibitors, degree of TR, presence of sinus node dysfunction, duration of cyanosis before the operation, BNP values, V˙O2max, CMR calculated mass of the morphologic systemic RV, RVEDV, VFrest, and VFdobutamine [CMR]	[50]
		MBFR	TAPSE, exercise capacity (% of predicted workload)	-	-	[43]
		MFR	-	-	Age, duration of follow-up since AtSO	[51]
	ccTGA	CFR	V˙O2max, function of the morphologic systemic RV (echocardiography), VFrest, and VFdobutamine (CMR)	BNP values, degree of TR, CMR calculated mass of morphologic systemic RV, RVEDV	ACE inhibitors, presence of sinus node dysfunction, duration of cyanosis before the operation	[50]
			V˙O2max, systolic ventricular function (echocardiography)	Age	Isolated = complex forms of ccTGA, mild = severe TR, ACE inhibitors, RV RPP calculated at rest and exercise, congenital heart block = regular sinus rhythm	[49]
**VSD**		CFVR	-	-	Qp/Qs	[53]
**CoA**		MPRI	-	-	Age, gender, tobacco use history, hypertension, medication use, age at repair, repair type, re-coarctation	[52]
		CFR	-	-	FMD, LVEF, LVIDd, BP	[56]

**Abbreviations:** ACE = angiotensin-converting enzyme, APV = average peak velocity, ASO = arterial switch operation, AtSO = atrial switch operation, BNP = brain natriuretic peptide, BP = blood pressure, CF(V)R = coronary flow (velocity) reserve, CMR = cardiac magnetic resonance, CoA = coarctation of the aorta, CPET = cardiopulmonary exercise testing, CS = coronary sinus, EF = ejection fraction, FMD = flow-mediated dilation, FS = fractional shortening, LAD = left anterior descending coronary artery, LV = left ventricular, LVIDd = LV internal diameter at end-diastole, M(B)FR = myocardial (blood) flow reserve, Qp/Qs = pulmonary-to-systemic flow ratio, RCA = right coronary artery, RVEDV = right ventricular end diastolic volume, TAPSE = Tricuspid annular plane systolic excursion, (cc)TGA = (congenitally corrected) transposition of the great arteries, TOF = tetralogy of Fallot, TR = tricuspid regurgitation, VF = ventricular function, V˙O2max = maximal oxygen consumption, VSD = ventricular septal defect.

* Number of banded patients too low for valid statistical analysis † Two patients with abnormal origin of the LAD had globally low MFR (<2.5).

## LIST OF ABBREVIATIONS

(cc)TGA: (congenitally corrected) Transposition of the great arteries
ACE: Angiotensin-Converting Enzyme
APV: Average Peak Velocity
AS: Aortic Stenosis
ASO: Arterial Switch Operation
AtSO: Atrial Switch Operation
BNP: Brain Natriuretic Peptide
BP: Blood Pressure
CCHD: Cyanotic Congenital Heart Disease
CFR: Coronary Flow Reserve
CFV: Coronary Flow Velocity
CFVR: Coronary Flow Velocity Reserve
CHD: Congenital Heart Disease
CMD: Coronary Microvascular Dysfunction
CMR: Cardiac Magnetic Resonance
CoA: Coarctation of the Aorta
CPET: Cardiopulmonary Exercise Testing
CRP: C-reactive Protein
CS: Coronary Sinus
CT(A): Computed Tomography (Angiography)
EF: Ejection Fraction
eNOS: Endothelial NO Synthase
FMD: Flow-Mediated Dilation
FS: Fractional Shortening
GCV: Great Cardiac Vein
HFpEF: Heart Failure with a Preserved Ejection Fraction
HLHS: Hypoplastic Left Heart Syndrome
IL: Interleukin
LAD: Left Anterior Descending Coronary Artery
LCA: Left Coronary Artery
LV: Left Ventricle/Ventricular
LVIDd: Left Ventricular Internal Diameter at End-Diastole
MBF: Myocardial Blood Flow
MBFR: Myocardial Blood Flow Reserve
MCE: Myocardial Contrast Echocardiography
MFR: Myocardial Flow Reserve
MPR(I): Myocardial Perfusion Reserve (Index)
NO: Nitric Oxide
NS: Not Specified
ONOO-: Peroxynitrite
PC VEC MRI: Phase-Contrast Velocity Encoding Cine Magnetic Resonance Imaging
PDA: Posterior Descending Coronary Artery
PET: Positron Emission Tomography
Qp/Qs: Pulmonary-to-Systemic Flow Ratio
RCA: Right Coronary Artery
ROS: Reactive Oxygen Species
RPP: Rate-Pressure Product
RV: Right Ventricle/Ventricular
RVEDV: Right Ventricular End-Diastolic Volume
sFas-ligand: Soluble Fas-Ligand
sICAM-1: Soluble Intercellular Adhesion Molecule-1
SPECT: Single-Photon Emission Computerized Tomography
sVCAM-1: Soluble Vascular Cell Adhesion Molecule-1
TAPSE: Tricuspid Annular Plane Systolic Excursion
TNFα: Tumor Necrosis Factor Alfa
TOF: Tetralogy of Fallot
TR: Tricuspid Regurgitation
TTE: Transthoracic Echocardiography
V’O2max: Maximal Oxygen Consumption
VF: Ventricular Function
VSD: Ventricular Septal Defect
